# A global visual method for measuring the deterioration of strawberries in MAP

**DOI:** 10.1016/j.mex.2018.07.012

**Published:** 2018-07-20

**Authors:** Céline Matar, Sébastien Gaucel, Nathalie Gontard, Stéphane Guilbert, Valérie Guillard

**Affiliations:** Joint Research Unit, Agropolymers Engineering & Emerging Technology, UM – INRA – Supagro & CIRAD, 2 Place Pierre Viala, Bat 31, 34060 Montpellier Cedex 01, France

**Keywords:** Global visual deterioration assessment of strawberries, Assessing deterioration, Deterioration grid, Texture softening, Color change, Microorganism development

## Abstract

Evaluating the quality changes of packed strawberries during storage requires multiple, time consuming and costly measurements such as sensorial, chemical and decay identification. In order to efficiently assess the quality of strawberries in Modified Atmosphere Packaging (MAP) while reducing the number of analysis done, we propose to gather the main visual quality changes under one unique, overall measurement. For this end, a protocol associated to a deterioration grid was built to evaluate surface deterioration as a function of time considering color change, texture softening and microorganism development. The developed method has permitted to build the deterioration kinetic of strawberries packed in different conditions (MAP or no MAP). It allows to mimic the quality analysis made by the consumer, at a glance, during purchase. To the best of our knowledge, the presented method is a breakthrough unlike most common usual methods mainly relying on the number of spoiled strawberries.

• Global measurement of the deterioration encompassing microorganism development, color change and texture softening.
• An annotation grid built to be used as reference for the attribution of the percentage of strawberries’ deterioration.
• Measurements of a percentage of surface deterioration was found more accurate than counting the number of rotten strawberries.

## Graphical abstract

**Figure:**
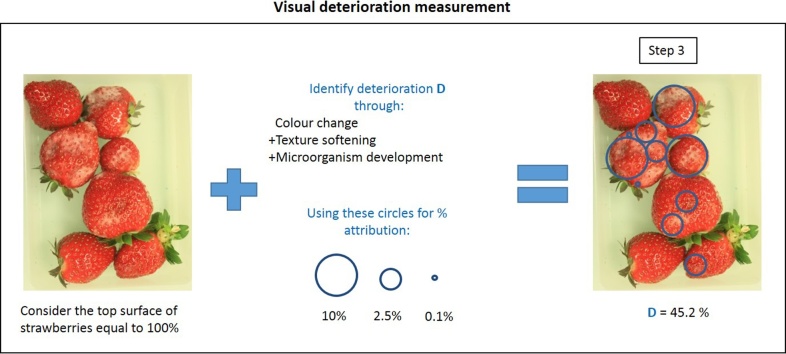

**Specifications Table**

**Table:** 

Subject area	*Agricultural and Biological Sciences*
More specific subject area	*Assessing product deterioration in MAP*
Method name	*Global visual deterioration assessment of strawberries*

## Methodology background

Assessing the quality of strawberries as a function of time is an important step for the characterization of products’ shelf life. So far, many quality parameters are measured in parallel to assess the overall quality of strawberries such as total soluble solids (TSS), firmness, color, decay on the fruit, etc. However, these multiple measurements are time consuming, require sophisticated equipment and a high amount of strawberries. In this paper, we present global visual evaluation of the strawberries’ quality expressed as the percentage of deteriorated surface area, namely percentage of deterioration. This visual percentage of deterioration encompass color change and/or texture softening and/or microorganism development, similarly to the analysis made by the consumer at purchase. In tight packed system, as in Modified Atmosphere Packaging, only visual assessment could be done. This methodology aims at mimicking this act of purchase.

## Method details

### Materials

- Strawberries of the variety “*Charlotte*”, grown off-ground, (Mauguio, South of France)
- Film packaging made of low density polyethylene (LDPE) (BBA emballages, Lunel – France)
- Polypropylene (PP) trays (Attitud’Pack, Chatuzange Le Goubet-France) with dimensions of 0.14 × 0.095 × 0.025 m
- Memmert incubators for controlling the temperature (Memmert, Schwabach – Germany)
- Digital camera for pictures acquisition (Canon camera EOS 450D – Japan)

### Method

#### Deterioration grid

A grid for measuring the percentage of fruit surface deterioration was built based on Ctifl studies [1,2]. The deterioration is expressed through a percentage of visual surface deterioration of the fruit.

During time, this percentage of surface deterioration will evolve with the visual aspect of the fruit due to the softening of the texture, colour change and development of microorganism on the surface. Only visual aspects were included while building the grid as it is the only way for a consumer to check the quality of the fruits packed in MAP for purchase decision.

The percentage of the deterioration is identified on the pictures using 3 circles of decreasing sizes as indicated in Table 1. The radius R of the big circle was set so that the area of 10 big circles represents the whole surface of the strawberries. Each big circle then corresponds to 10% of deteriorated surface. In order to have a more accurate estimation of the deterioration, medium and small circles were also considered with radius R/2 and R/10 and associated percentages of deterioration equal to 2.5% and 0.1% respectively.

**Table 1:** Circles sizes used to estimate the percentage of deterioration.

Size of the circle		% of surface deterioration
Big		D_BC_ = 10 %
Medium		D_MC_ = 2.5 %
Small		D_SC_ = 0.1 %

To estimate a percentage of deterioration, the large damaged surfaces are first identified using big circles followed by lower damaged surfaces using medium and small ones. Note that all circles should be pairwise disjoint i.e. without intersection.

**Fig. 1:**
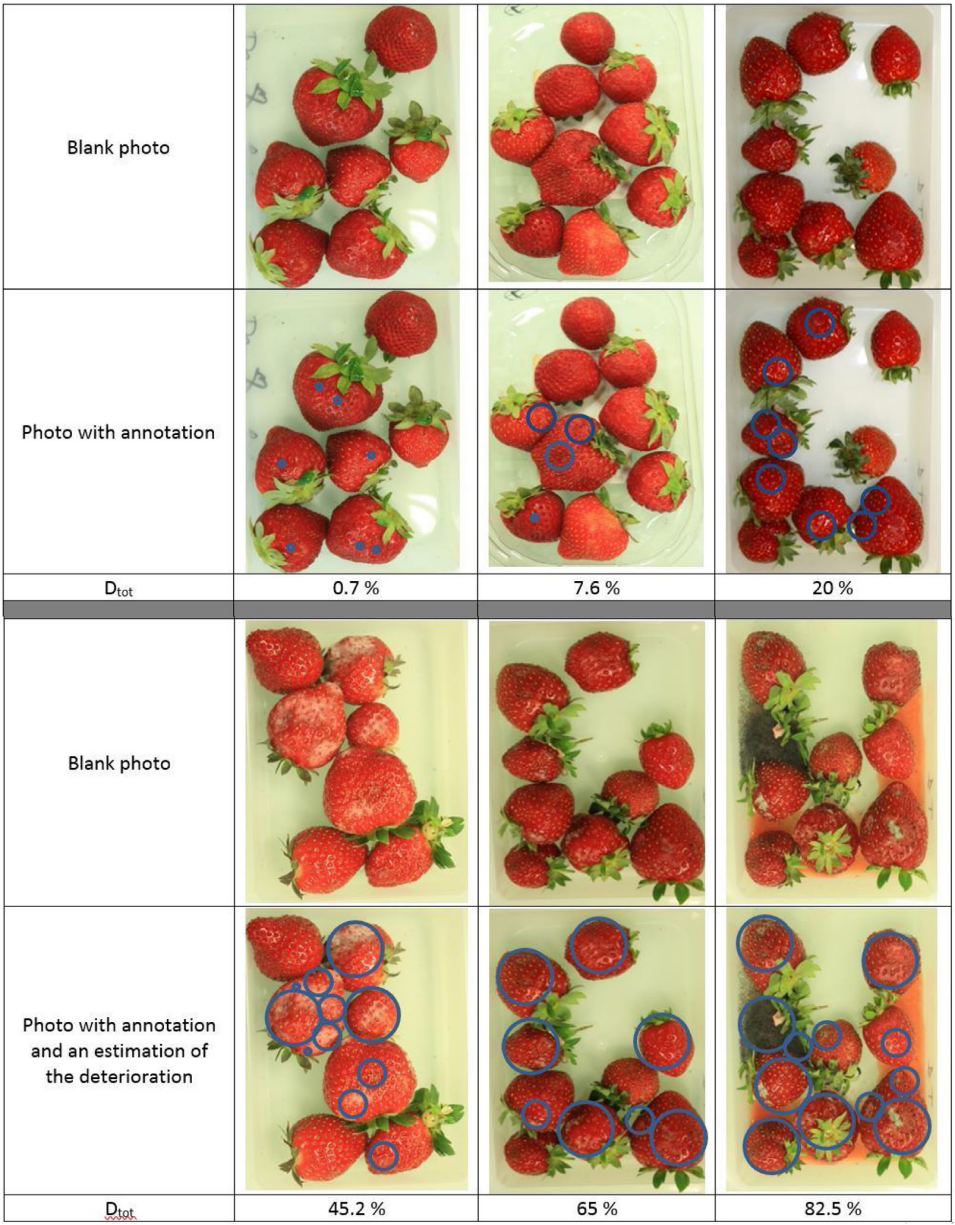
Examples of deterioration annotation using small circles correspond to 0.1% of deterioration, medium circles to 2.5% of deterioration and big circles to 10% of deterioration.

The total percentage of deterioration D_tot_ (%) is expressed through the following equation:

(1) $$\text{D}\text{tot}\text{ =}\;{\text{n}}_{\text{BC}}\;{\text{D}}_{\text{BC}}\;+\;{\text{n}}_{\text{MC}}\;{\text{D}}_{\text{MC}\;}+\;{\text{n}}_{\text{SC}}\;{\text{D}}_{\text{SC}}$$

with n_BC_ the number of big circles, n_MC_ the number of medium circles, n_sc_ the number of small circles, D_BC,_ D_MC_ and D_sc_ the percentage of deterioration associated respectively to the big, the medium and the small circles. Examples of annotation are given in Fig. 1 for different levels of deterioration.

#### Experimental design

1 Strawberries were harvested in the morning of experiments, cooled to 5 °C at the producer facilities, then picked up to the laboratory around 5 h after harvest where they were stored at desired temperatures for 3 h.
2 Harvested strawberries were sorted according to maturity: over ripened i.e. fruits with soft texture, damaged or rotten were eliminated and under ripened fruits i.e. fruits with green, not fully mature, spots were discarded. Sorting was also done according to shape: oversized fruits or very small ones compared to the batch fruit sizes were eliminated.
3 In total 42 trays of 100 g of strawberries were prepared, 6 trays of strawberries were packed in macro perforated LDPE and were used as the control situation representing no MAP condition and 36 were put in MAP conditions at the same time t = 0 using a non-perforated LDPE film packaging of 0.03 × 0.15 × 0.11 m dimensions and 50.5 × 10^6^ m thickness (Table 2).

**Table 2:** Experimental plan indicating the label of the tray analyzed at the corresponding days, for measurement done in MAP and no MAP conditions in the morning and in the afternoon.

	Storage condition						
Number of the measurement	Control (no MAP)	MAP					
		Day 1	Day 2	Day 3	Day 4	Day 5	Day 6
1st (Morning)	Tray 1	Tray 7	Tray 13	Tray 19	Tray 25	Tray 31	Tray 37
	Tray 2	Tray 8	Tray 14	Tray 20	Tray 26	Tray 32	Tray 38
	Tray 3	Tray 9	Tray 15	Tray 21	Tray 27	Tray 33	Tray 39

2nd (Afternoon)	Tray 4	Tray 10	Tray 16	Tray 22	Tray 28	Tray 34	Tray 40
	Tray 5	Tray 11	Tray 17	Tray 23	Tray 29	Tray 35	Tray 41
	Tray 6	Tray 12	Tray 18	Tray 24	Tray 30	Tray 36	Tray 42
4 A first measurement of the deterioration using the grid was done for the 36 trays at t = 0 right before packing the product in MAP conditions.
5 After t = 0, every measurement of the deterioration in MAP is a destructive one. Indeed, the estimation of the percentage of surface deterioration was done after removing the film packaging, to be able to see clearly the surface of the fruit and thus after breaking the MAP condition. In consequence, each one of the 36 trays in MAP condition were for single-use. However, for no MAP condition, the same 6 trays were assessed for the whole duration of the experiment since the gases surrounding the product is always equal to the atmosphere composition and will not affect the deterioration of the product (Table 2).
6 Measurements of the deterioration is done twice a day, in the morning and in the afternoon during 6 days, for MAP and no MAP conditions. For each measurement, three replicates, corresponding to three trays, were evaluated (Table 2).

Note that, a picture of the opened trays is taken right after breaking the MAP condition and will later be examined and compared to the percentage of deterioration identified in the deterioration grid (Fig. 1).

7 In parallel, a survey was conducted to identify the time at which the consumer will stop purchasing the product. For this end, a panel of 30 untrained consumers annotated the trays used for deterioration assessment, twice a day in the morning and in the afternoon for 6 days. The question asked to each consumer of this panel was ‘Just by looking at the strawberries in the tray, are you willing to buy the product or not?’ the 2 possible answers are ‘Yes’ or ‘No’. When more than 50% of the consumer answered ‘No’, the product is considered not marketable anymore.

#### Method validation

In order to validate the visual evaluation of the strawberries’ deterioration in MAP, we compared it to the common visual decay method used in the literature. The aforementioned method is based on a visual measurement of the % of decay or spoilage i.e. by counting the number of strawberries showing visible mold lesions on the surface expressed as a percentage of the total number of strawberries in the tray [[3], [4], [5]]. Decay is thus expressed in percentage as follow:

(2) $$\text{S}\;\text{=}\;\text{(}{\text{n}}_{\text{infec}}\;\times \;\text{100)/}{\text{n}}_{\text{tot}}$$

where S is the decay in %, n_infec_ is the number of molded strawberries in the package, n_tot_ is the total number of strawberries in the package.

The evaluation of decay (based on counting spoiled strawberries [[3], [4], [5]]) and deterioration (based on surface (method developed in this article)) were conducted simultaneously, on the same samples using the experimental plan explained in Experimental design section, for strawberries packed in MAP and no MAP conditions at 20 °C.

**Fig. 2:**
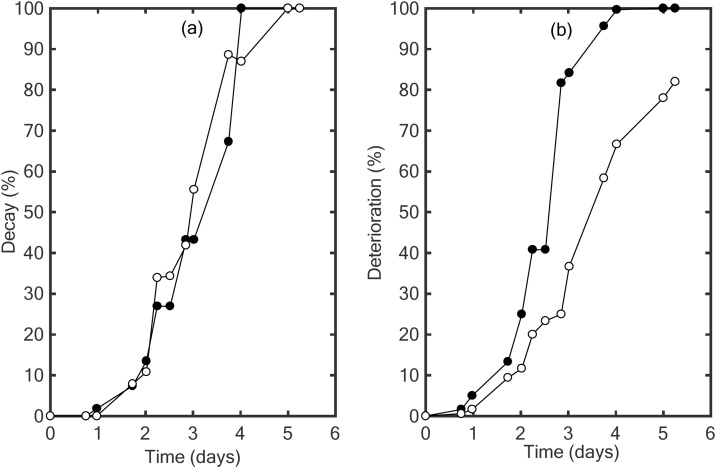
Visual assessment of a percentage of decay (a) using method from the literature and a percentage of deterioration (b) using methodology presented in the current manuscript, as a function of time in days, for strawberries packed in MAP (**○**) and no MAP condition (**●**) at 20 °C.

Experimental values of decay (Fig. 2(a)) and deterioration (Fig. 2(b)) in MAP (**○**) or no MAP (**●**) conditions as a function of time showed a sigmoidal behavior from 0% to 100% of decay or deterioration.

However, in Fig. 2(a) the experimental points in MAP (**●**) and no MAP (**○**) for decay lead to quasi superposition of the two sigmoidal curves, depicting the same behavior in MAP and no MAP condition. Therefore, differentiation between MAP and no MAP is difficult to assess. Thus, annotation based on a percentage of decay was not sufficient on its own in the identification of the quality difference between MAP and no MAP results. In fact, decay was identified through the number of molded strawberries in both MAP and no MAP conditions but no discrimination was made on the level of fruit’s decay.

Whereas, in Fig. 2(b), the percentage of deterioration in MAP and no MAP are separated in two independent sigmoidal curves. MAP condition result in a slight inhibition of the deterioration compared to no MAP condition. At 5.25 days, 100% of the strawberries packed in no MAP are affected as compared to 82% of strawberries in MAP condition. Unlike decay method, the deterioration grid was able to differentiate between MAP and no MAP condition because the deterioration method takes into account the surface of the fruit affected by a texture softening and/or microorganism development and/or color change at the same time.

Results of the survey showed that trays in no MAP conditions were not marketable from day 2 (morning) where more than 50% of the consumers answered ‘No’ to the asked question. However, trays in MAP condition were judged acceptable by the consumers until day 3 (morning). Therefore, consumer survey confirms that the kinetic of degradation of strawberries is slower in MAP condition than in no MAP. This difference of behavior of strawberries quality in two storage conditions, here MAP and no MAP, is well described by the deterioration factor while decay factor leads to a contradictory conclusion.
